# Microbiota-Macroalgal Relationships at a Hawaiian Intertidal Bench Are Influenced by Macroalgal Phyla and Associated Thallus Complexity

**DOI:** 10.1128/mSphere.00665-21

**Published:** 2021-09-22

**Authors:** Gabrielle M. Kuba, Heather L. Spalding, Kristina M. Hill-Spanik, Heather Fullerton

**Affiliations:** a Department of Biology, College of Charleston, Charleston, South Carolina, USA; b Department of Biology, Grice Marine Lab, College of Charleston, Charleston, South Carolina, USA; Clemson University

**Keywords:** microbiome, macroalgae, invasive algae, native algae

## Abstract

The ocean represents the largest biome on earth; however, we have only begun to understand the diversity and function of the marine microbial inhabitants and their interactions with macroalgal species. Macroalgae play an integral role in overall ocean biome health and serve both as major primary producers and foundation species in the ecosystem. Previous studies have been limited, focusing on the microbiome of a single algal species or its interaction with selected microbes. This project aimed to understand overall biodiversity of microbial communities associated with five common macroalgal species and to determine the drivers of these communities at ‘Ewa Beach, O‘ahu, HI. Representative species of Chlorophyta (green), Ochrophyta (brown), and Rhodophyta (red) algae, each species having various levels of calcification, thallus complexity, and status as native or invasive species, were collected from an intertidal bench in May 2019. A portion of the V3-V4 variable region of the small-subunit rRNA gene was amplified for high-throughput sequencing using universal bacterial primers to elucidate the core and variable algal microbiome. Significant differences in bacterial community composition were only partially explained by host species, whether the host was native or invasive, and thallus complexity. Macroalgal phylum explained the most variation in associated microbial communities at ‘Ewa Beach. This study advances our understanding of microbial-macroalgal interactions and their connectivity by producing insight into factors that influence the community structure of macroalga-associated microbiota.

**IMPORTANCE** Generally, most eukaryotic organisms form relationships with microbes that are important in mediating host organismal health. Macroalgae are a diverse group of photosynthetic eukaryotic organisms that serve as primary producers and foundational species in many ecosystems. However, little is known about their microbial counterparts across a wide range of macroalgal morphologies, phylogenies, and calcification levels. Thus, to further understand the factors involved in bacterial community composition associated with macroalgal species at one point in time, representative samples were collected across phyla. Here, we show that both host macroalga phyla and morphology influenced the associated microbial community. Additionally, we show that the invasive species Avrainvillea lacerata does not have a unique microbial community on this intertidal bench, further supporting the idea that host phylum strongly influences microbial community composition.

## INTRODUCTION

Microorganisms are ubiquitous throughout the environment and form relationships with larger eukaryotic organisms that are important in mediating host health (1, 2). Bacterial community composition can be influenced by multiple aspects of the eukaryotic host, such as biogeography (3, 4), morphological niche (3, 5–9), health (10, 11), and morphological complexity (1, 6, 12–14). These factors can act independently or in association with one another and can vary on an individual level (15).

One group of eukaryotic hosts of interest is macroalgae, a morphologically and taxonomically diverse photosynthetic group of organisms that serve as major primary producers and foundational species within ecosystems (16–19). Additionally, Rhodophyta, Ochrophyta, and Chlorophyta are often found inhabiting the same intertidal and photic zones (20, 21). Previous studies have identified specific functions that microbes perform when in association with their hosts (22). These roles include exchange of nitrogen (23–25), detoxification of pollutants (23, 24), the production of secondary metabolites that are directly and indirectly linked to the host functionality (11, 14, 23, 26), development of host morphology (12), and the production of essential vitamins such as B_12_ (27, 28).

Marine bacteria organize into biofilms that form a secondary skin on the macroalgal host. This biofilm can influence nutrient uptake and the production of specific chemical cues related to identification and recognition of the host by other flora and fauna (19, 29–31). In addition to performing certain functions themselves, associated microbiota can encourage specific macroalgal host functions such as signal transduction and gene transfer (32), growth stimulation (1, 33, 34), morphogenesis (12), spore germination (35), nitrogen metabolism (1), and antifouling defense (32, 36). Macroalgal hosts can alter their associated microbiota, selecting for counterparts that are beneficial to their survival (29). For example, healthy Gracilaria conferta (Rhodophyta) controls its epibiotic colonization through the production of chemical signals (32, 37), utilizing the production of specific surface metabolites to attract protective bacteria or deter pathogenic strains (29).

**FIG 1:**
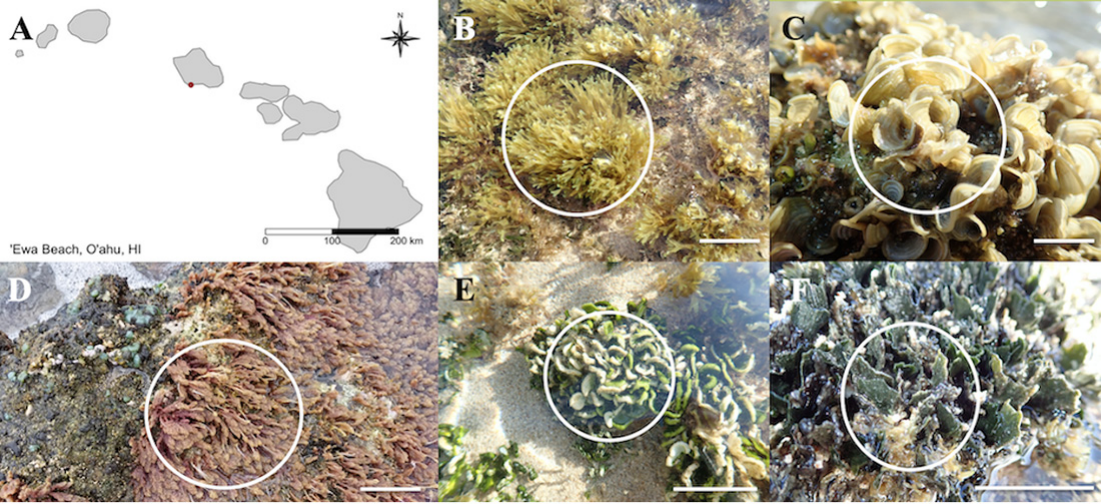
Map of sample site at ‘Ewa Beach, O‘ahu, HI, USA (A), and morphological identification of macroalgal species in this study: *Dictyota sandvicensis* (B), *Padina sanctae-crucis* (C), *Asparagopsis taxiformis* (D), *Halimeda discoidea* (E), and *Avrainvillea lacerata* (F). Bars, 5 cm.

‘Ewa Beach, O‘ahu, HI, USA, is culturally and historically unique, serving as a collection site of macroalgae, or limu, for local residents. This beach is characterized by a series of rocky intertidal benches interspersed with sand, which provides a hard substrate for macroalgal attachment (Fig. 1A). ‘Ewa Beach has historically been impacted by anthropogenic influences, such as nutrient influxes from sewage and sugarcane-based agriculture, that affect long-term ecosystem variations (21, 38, 39). Previous studies have provided descriptions of macroalgal diversity and the processes that influence their structure both spatially and temporally (21, 38). Macroalgal diversity at this site had been impacted by both abiotic and biotic factors, specifically, temperature increases and the invasive alga Avrainvillea lacerata (J. Agardh), formerly Avrainvillea amadelpha (21, 40). *A. lacerata* was first identified in Hawaiian subtidal zones in the 1980s (41) and since then has expanded its range into the intertidal coastal waters (21, 42). This species has also been observed in high abundance at mesophotic depths (to 90 m) around western and southern O‘ahu (43). At ‘Ewa Beach, *A. lacerata* increased in abundance from <1% cover in 2012 (21) to 25 to 50% current cover by 2021 in the intertidal waters (H. L. Spalding, personal observations).

To characterize the diversity of macroalgal-associated microbiota, previous research focused on a subset of the microbial community on a single algal genus using culture-dependent techniques (14, 44–46) or, more recently, using culture-independent analyses of the total microbiota (3, 47). In culture-independent analyses, high-throughput DNA amplicon sequencing enables a more comprehensive look at total bacterial community. These types of analyses typically use the highly conserved small-subunit (SSU) rRNA gene because small variations in the gene can indicate large evolutionary distances (48). The associated analyses typically begin with the construction of operational taxonomic units (OTUs), with SSU rRNA gene sequences clustered based on a 97% similarity threshold, which corresponds to bacterial species level identification (49). Recently, the divisive amplicon denoising algorithm (DADA2) was developed (50). DADA2 defines amplicon sequence variants (ASVs) on the basis of error-corrected nucleotide differences and identifies sequence variants from a sample more accurately than OTU-picking algorithms (50, 51).

In this study, the most abundant species of Chlorophyta, Ochrophyta, and Rhodophyta from ‘Ewa Beach, O‘ahu, HI, were used to examine the microbial-macroalgal diversity. To allow detailed analyses of the host factors as drivers of bacterial community structure, rather than environmental or temporal variations, all samples were collected from the same intertidal bench at the same time. Using culture-independent techniques with ASV identification, this study hypothesized that host phylum influences the microbiota diversity associated with five species of macroalgae. Additionally, because ‘Ewa Beach was recently invaded by *A. lacerata*, this species was hypothesized to have a distinct microbial community compared to the native macroalgae. Other factors, such as thallus complexity and phylogenetic affinities, were hypothesized to have less influence on the macroalga-associated microbiota diversity.

## RESULTS

### Sample collection and macroalgal characterization.

The five most abundant macroalgae at ʻEwa Beach were collected: two species of Chlorophyta (Halimeda discoidea and *Avrainvillea lacerata*), two species of Ochrophyta (Padina sanctae-crucis and Dictyota sandvicensis), and one species of Rhodophyta (Asparagopsis taxiformis) (Fig. 1). Key characteristics of these species are noted in Table 1. The calcification levels described include uncalcified, lightly calcified, and calcified. Thallus characteristics of each species were classified based on previously described observations (52, 53).

**TABLE 1:** Macroalgal samples (*n* = 3 per sample) and their associated characteristics^*a*^

Samples	Host species	Macroalgal phylum	Invasive/native	Calcification level	Thallus complexity
Pa.sa1, Pa.sa2, Pa.sa3	*Padina sanctae-crucis*	Ochrophyta	Native	Lightly calcified	Fan-shaped thallus
Di.sa1, Di.sa2, Di.sa3	*Dictyota sandvicensis*	Ochrophyta	Native	Uncalcified	Flattened dichotomous branches
Ha.di1, Ha.di2, Ha.di3	*Halimeda discoidea*	Chlorophyta	Native	Calcified	Flattened segments
Av.la1, Av.la2, Av.la3	*Avrainvillea lacerata*	Chlorophyta	Invasive	Uncalcified	Fan-shaped thallus
As.ta1, As.ta2, As.ta3	*Asparagopsis taxiformis*	Rhodophyta	Native	Uncalcified	Filamentous upright axes

a Samples were collected from the intertidal zone at ʻEwa Beach, Oʻahu, HI, USA (21.3058 N, 158.0284 W).

### Microbial characterization.

**FIG 2:**
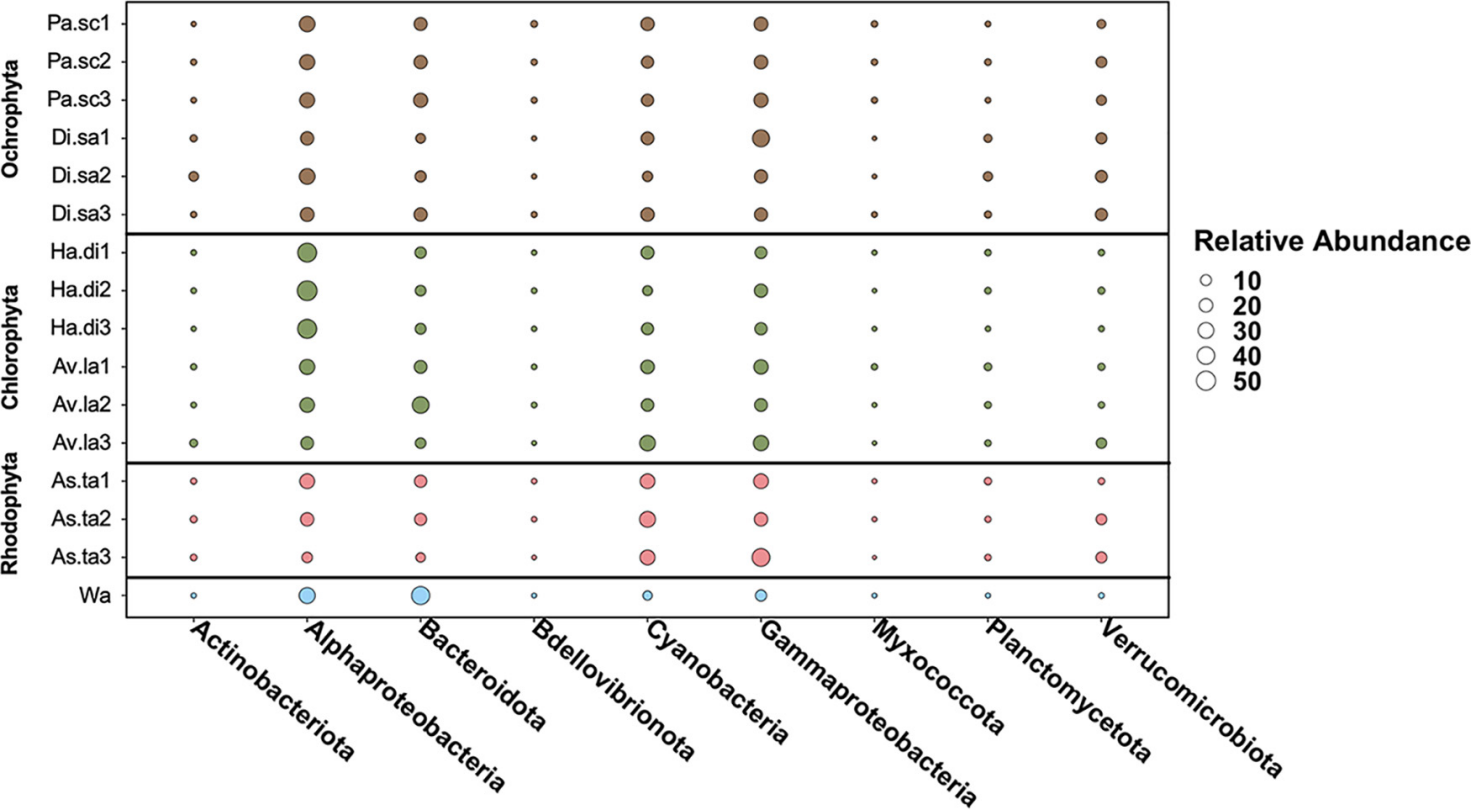
Taxonomic distribution of associated bacteria genera of the top 3% in relative abundance. The relative abundances of bacteria phyla are provided for each sample. The associated colors correspond to the sample type: brown, Ochrophyta; green, Chlorophyta; pink, Rhodophyta; blue, water control. Each sample (*n* = 15) is shown except for seawater control samples (*n* = 3), which were pooled prior to indexing and sequencing. Sample identification corresponds to the given species: Pa.sa, *Padina sanctae-crucis*; Di.sa, *Dictyota sandvicensis*; Ha.di, *Halimeda discoidea*; Av.la, *Avrainvillea lacerata*; As.ta, *Asparagopsis taxiformis*; Wa, Seawater control.

A total of 15 macroalgal samples and one water control were sequenced, resulting in 4,447,308 total reads with an average length of 414 ± 15 bp after quality control and filtering (Table S3). Initially, the total number of ASVs was 44,160. After chimera identification, the final ASV number was 26,257. The relative abundances for algal individuals were not averaged due to variability observed among replicates. *Alphaproteobacteria*, *Bacteroidota*, *Cyanobacteria*, *Gammaproteobacteria*, and *Verrucomicrobiota* were the most abundant bacterial taxa across all macroalgal phyla (Fig. 2; Fig. S2 and S4). Additionally, *Actinobacteriota*, *Bdellovibrionota*, *Myxococcota*, and *Planctomycetota* were found to be associated in higher abundance with the ochrophytes (Fig. 2; Fig. S4).

10.1128/mSphere.00665-21.2 FIG S2 Stacked bar chart showing the percentage of 16S rRNA gene copies recovered for major bacterial taxa across representative (A) Chlorophyta, (B) Ochrophyta, and (C) Rhodophyta samples. Download FIG S2, PDF file, 0.6 MB. Copyright © 2021 Kuba et al. 2021 Kuba et al. https://creativecommons.org/licenses/by/4.0/ This content is distributed under the terms of the Creative Commons Attribution 4.0 International license.

10.1128/mSphere.00665-21.4 FIG S4 Heat map of the taxonomic distribution of macroalgal-associated bacteria genera of the top 3% in relative abundance. The relative abundances of bacteria phyla are provided for each sample. Each macroalgal phylum is considered (Ochrophyta, Chlorophyta, Rhodophyta), as well as the background seawater control. Individual samples (*n* = 15) are shown except for seawater control samples (*n* = 3), which were pooled prior to indexing and sequencing. Sample identification corresponds to the given species: Pa.sa, *Padina sanctae-crucis*; Di.sa, *Dictyota sandvicensis*; Ha.di, *Halimeda discoidea*; Av.la, *Avrainvillea lacerata*; As.ta, *Asparagopsis taxiformis*; Wa, Seawater control. Download FIG S4, PDF file, 0.5 MB. Copyright © 2021 Kuba et al. 2021 Kuba et al. https://creativecommons.org/licenses/by/4.0/ This content is distributed under the terms of the Creative Commons Attribution 4.0 International license.

10.1128/mSphere.00665-21.7 TABLE S3 Read counts throughout input, trimming, quality check, and chimera identification for each of the samples. Download Table S3, PDF file, 0.2 MB. Copyright © 2021 Kuba et al. 2021 Kuba et al. https://creativecommons.org/licenses/by/4.0/ This content is distributed under the terms of the Creative Commons Attribution 4.0 International license.

*Verrucomicrobiota* were found in higher relative abundance in all species within the Ochrophyta and Rhodophyta than the Chlorophyta (Fig. S2 and S4). *A. taxiformis* (Rhodophyta) was characterized mainly by the same bacterial taxa as the other two macroalgal phyla (Fig. 2; Fig. S2). However, *D. sandvicensis* (Ochrophyta) had a higher relative abundance of *Actinobacteriota* and *Planctomycetota* than *P. sanctae-crucis* (Ochrophyta). The relative abundances of bacterial taxa associated with the Chlorophyta were similar to those associated with the Ochrophyta (Fig. 2); one of the main differences between these species was that *A. lacerata* had a greater relative abundance of *Verrucomicrobiota* than *H. discoidea* and had representatives of *Actinobacteriota* and *Myxococcota* (Fig. S4).

The background seawater microbial community had representative bacteria across taxa similar to those in the macroalgal samples, including *Alphaproteobacteria*, *Bacteroidota*, *Cyanobacteria*, and *Gammaproteobacteria* in relative abundances similar to those in the macroalgal species. These ASVs were removed in the subsequent core microbiome analyses (see below). However, there were no representatives of the *Actinobacteriota*, *Planctomycetota*, *Myxococcota*, and *Verrucomicrobiota* in the seawater control, suggesting that these bacterial taxa form associations with the algae (Fig. 2).

The total observed ASVs ranged from 1,457 (*D. sandvicensis* [Di.sa1]) to 8,882 (*H. discoidea* [Ha.di1]) (Table 2). Species richness rarefaction curves showed that each curve reached a plateau, suggesting adequate microbial community representation (Fig. S1). Of the observed values within each species, the measurements were variable, with the highest being associated with *H. discoidea. A. lacerata* had the lowest diversity as described by the Simpson index, followed by *H. discoidea*, *A. taxiformi*s, *D. sandvicensis*, and *P. sanctae-crucis*. Additionally, the microbial counterparts associated with Ochrophyta and both Chlorophyta and Rhodophyta individuals were significantly different, with *P* values of 0.013 and 0.004, respectively (Table S4).

**TABLE 2:** Observed ASVs of bacterial taxa and diversity indices of the microbial communities associated with macroalgal species

Sample type	Sample name	Observed ASVs	Diversity index^*a*^	
			Shannon	Simpson
Ochrophyta	Pa.sc1	2,680	2.65	0.53
	Pa.sc2	2,932	2.64	0.56
	Pa.sc3	2,472	1.88	0.38
	Di.sa1	1,457	2.17	0.52
	Di.sa2	2,424	2.73	0.58
	Di.sa3	2,877	2.94	0.66
Chlorophyta	Ha.di1	3,248	5.35	0.98
	Ha.di2	3,443	4.95	0.97
	Ha.di3	3,766	5.12	0.97
	Av.la1	8,882	7.18	0.99
	Av.la2	4,787	5.73	0.97
	Av.la3	2,518	4.88	0.93
Rhodophyta	As.ta1	3,525	6.33	0.99
	As.ta2	4,180	5.76	0.96
	As.ta3	2,009	3.69	0.83
Water control	Wa	2,340	5.34	0.98

a Shannon-Weiner and Simpson indices were chosen to encompass the variability in both abundance and evenness of ASVs seen across macroalgal samples.

10.1128/mSphere.00665-21.1 FIG S1 Rarefaction curves of bacterial partial small-subunit (SSU) rRNA gene sequences for macroalgal samples and combined water control. Macroalgal phyla are indicated by colors: Chlorophyta (green), Ochrophyta (brown), Rhodophyta (red). Water control (blue) is also included. The sampling depth associated with the fewest sequences is indicated by the black line. Download FIG S1, PDF file, 0.7 MB. Copyright © 2021 Kuba et al. 2021 Kuba et al. https://creativecommons.org/licenses/by/4.0/ This content is distributed under the terms of the Creative Commons Attribution 4.0 International license.

10.1128/mSphere.00665-21.8 TABLE S4 PERMANOVA results based on Bray-Curtis dissimilarities of amplicon sequence variant abundances for bacterial communities within the complete macroalgal microbiota. Comparisons were made between microbial counterparts associated with specific macroalgal phyla. An asterisk indicates a significant associated *P* value; this value indicates a difference between group dispersions in the betadisper test. Download Table S4, PDF file, 0.01 MB. Copyright © 2021 Kuba et al. 2021 Kuba et al. https://creativecommons.org/licenses/by/4.0/ This content is distributed under the terms of the Creative Commons Attribution 4.0 International license.

Ochrophyta had the highest diversity of associated microbiota by the Simpson index compared to both Chlorophyta and Rhodophyta (Table 2), similar to previous findings (Table 2) (54, 55). Despite the high associated microbial diversity, ochrophytes had the lowest associated observed microbial ASVs (Table 2; Table S2). Chlorophyta species had relatively high observed microbial ASVs but low microbial diversity (Table 2; Table S2). Interestingly, the invasive *A. lacerata* possessed the highest number of observed ASVs but the lowest associated diversity. The representative Rhodophyta species had observed microbial ASVs comparable to those seen with the native Chlorophyta species (*H. discoidea*) and both Ochrophyta but again had low microbial diversity (Table 2; Table S2). As expected, the combined water control was associated with low numbers of observed microbial ASVs and diversity compared to the macroalgal phyla (Table 2; Table S2).

10.1128/mSphere.00665-21.6 TABLE S2 Observed ASVs of bacterial taxon diversity indices of the microbial communities associated with macroalgal species. Diversity indices calculated include Chao1, abundance-based coverage estimator (ACE), Shannon-Weiner, Simpson, inverse Simpson (InvSimpson), and Fisher. Download Table S2, PDF file, 0.02 MB. Copyright © 2021 Kuba et al. 2021 Kuba et al. https://creativecommons.org/licenses/by/4.0/ This content is distributed under the terms of the Creative Commons Attribution 4.0 International license.

### Core microbiome of dominant macroalgae on ‘Ewa Beach.

**FIG 3:**
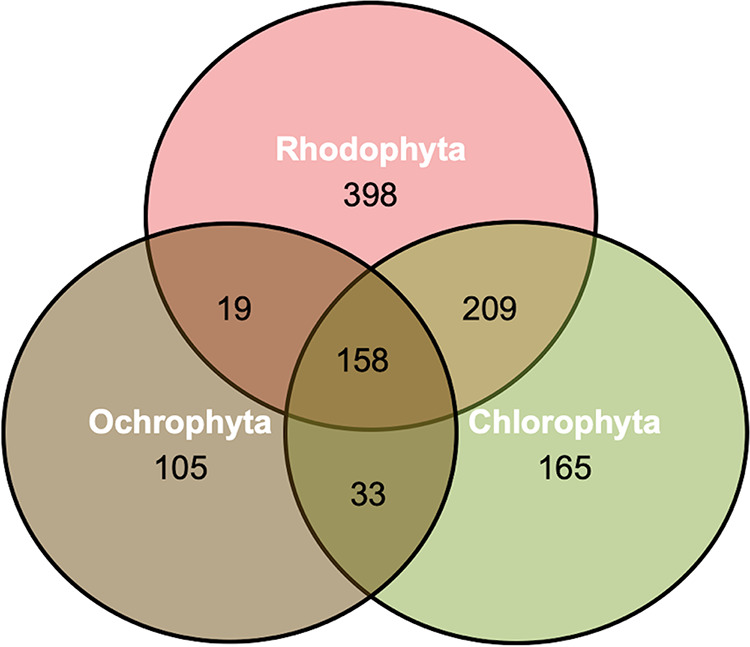
Venn diagram showing the numbers of unique and shared amplicon sequence variants among the phyla of macroalgae.

To identify the core microbial ASVs associated with these macroalgal species, ASVs found within the background water control were removed. This analysis identified 158 ASVs in common across all five species of algae. Ochrophytes had the least microbial overlap compared to other taxonomic groups, sharing only 19 taxa with the rhodophytes and 33 with the chlorophytes (Fig. 3), whereas the rhodophytes and chlorophytes shared 209 microbial ASVs. The chlorophytes and rhodophytes also had more unique ASVs, with 165 and 398, respectively. Although ochrophytes had the highest diversity, they contained the lowest number of unique ASVs (105) (Fig. 3).

Analysis of the most abundant shared microbial genera among all three macroalgae phyla was variable. The bacteria *Acrophormium* PCC 737, *Hyphomonas*, *Rivularia* PCC 7116, and *Schizothrix* 07164 were found across all macroalgal phyla. *Rivularia* PCC 7116 was found in higher relative abundance in the Ochrophyta and Chlorophyta than in the Rhodophyta (Table S5). *Schizothrix* 07164 was found in greatest relative abundance in the Chlorophyta and the lowest in the Rhodophyta. *Acrophormium* PCC 737 was found in highest relative abundance in association with the Rhodophyta and had lower relative abundance in the Chlorophyta and Ochrophyta. *Hyphomonas* was the second most relatively abundant bacterial genus across all macroalgal phyla (Table S5). Overall, there was one genus unique to each macroalgal phyla: *Litorimonas* sequences were found as the third most relatively abundant bacterial genus associated with Ochrophyta, *Cognatishimia* sequences with Rhodophyta, and *Limibaculum* sequences with Chlorophyta (Table S5).

10.1128/mSphere.00665-21.9 TABLE S5 The five most abundant bacterial genera associated with each macroalgal phylum (Ochrophyta, Rhodophyta, and Chlorophyta) at ‘Ewa Beach, Hawai’i, USA. Download Table S5, PDF file, 0.06 MB. Copyright © 2021 Kuba et al. 2021 Kuba et al. https://creativecommons.org/licenses/by/4.0/ This content is distributed under the terms of the Creative Commons Attribution 4.0 International license.

### Influence of phyla on macroalga-associated microbiota.

**FIG 4:**
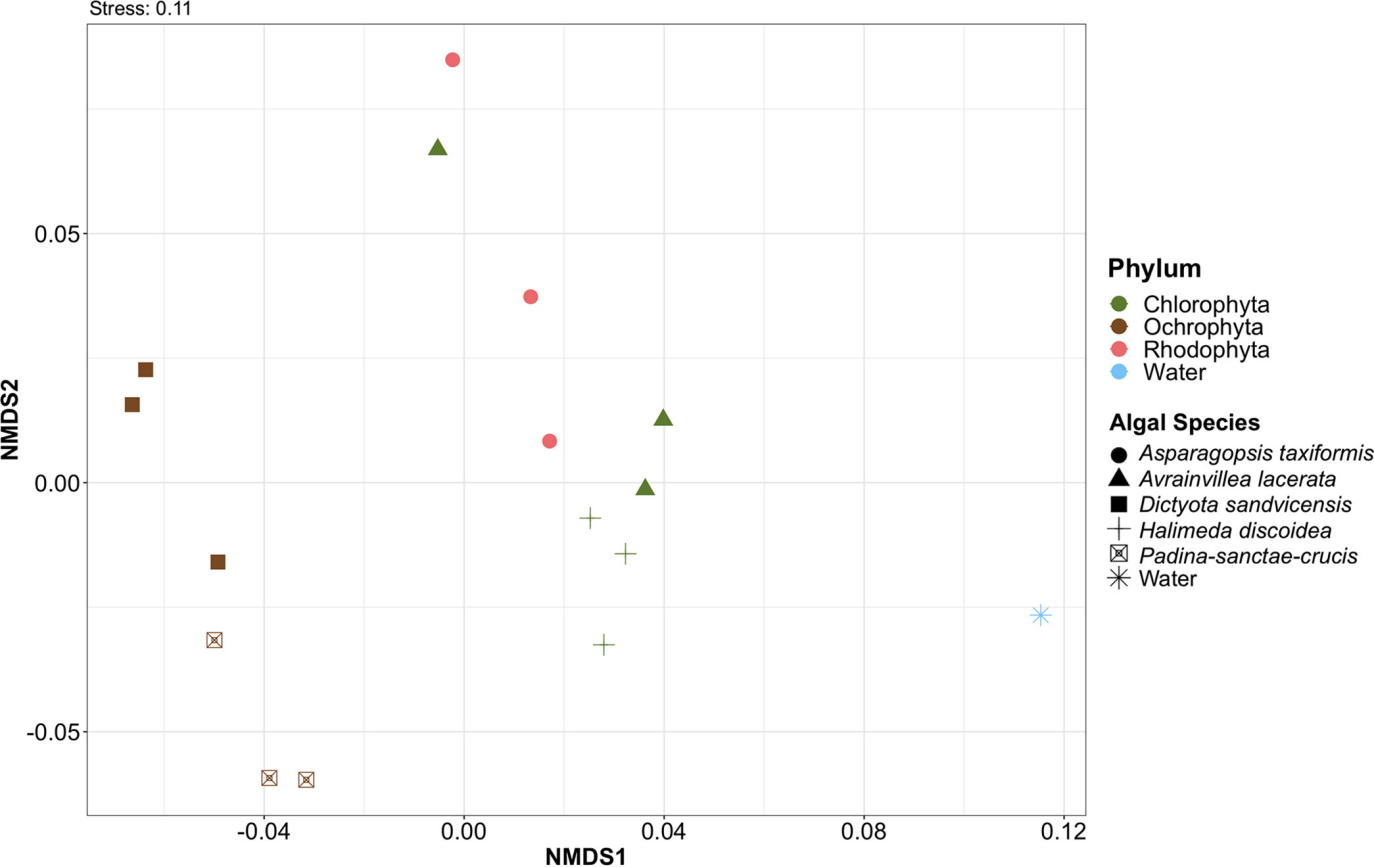
Pearson correlation nonmetric multidimensional scaling (NMDS) plot generated using Bray-Curtis dissimilarities for microbial communities associated with macroalgal species. The stress level associated with these assemblages is 0.11. *Avrainvillea lacerata* is an invasive species at this Hawaiian intertidal bench, whereas all other species are native. Individual samples (*n* = 15) are shown, except for seawater control samples (*n* = 3), which were pooled prior to sequencing.

Community comparisons between macroalgae were completed to analyze the influence of macroalgal phyla and the native/invasive classification of Chlorophyta on the associated ASVs. The nonmetric multidimensional scaling (NMDS) plot showed a distinction between the Ochrophyta and other algal phyla (Fig. 4), as expected based on number of unique ASVs. The native Chlorophyta species (*H. discoidea*) was more tightly clustered than the invasive (*A. lacerata*), while *A. lacerata* overlapped with *A. taxiformis* (Rhodophyta). This suggests that the microbiota associated with *A. lacerata* are influenced more by other factors than algal phylum. The NMDS and permutational multivariate analysis of variance (PERMANOVA) results based on Bray-Curtis dissimilarities were in general agreement with hierarchical clustering based on Euclidean distances (Fig. S3). The Ochrophyta were clustered tightly, whereas the Rhodophyta were interspersed throughout the Chlorophyta clustering (Fig. S3). The native Chlorophyta *H. discoidea* individuals clustered closely, while the invasive *A. lacerata* possessed a more sporadic clustering pattern (Fig. S3). Although there were differences in associated microbiota between *H. discoidea* and *A. lacerata*, these differences were not significant based on PERMANOVA (Table 3; Table S6).

**TABLE 3:** Summary of PERMANOVA based on Bray-Curtis dissimilarities of ASV abundances for bacterial communities within the complete microbiota and group-level microbial counterparts

Factor	Sums of squares	Mean square	*F* value	*R* ^2^	*P* value^*a*^
Phylum	340,735	170,367	2.165	0.265	**0.003**
Host species	575,092	143,773	2.026	0.448	**0.001**
Invasive/native	146,409	146,409	1.672	0.114	0.052
Thallus complexity	379,521	126,507	1.537	0.295	**0.017**
Calcification of Chlorophyta^*b*^	131,547	131,547	1.401	0.259	0.100
Calcification of Ochrophyta^*b*^	102,811	102,811	2.171	0.352	0.100

a Boldface indicates a significant difference in community composition.

b Calcification level was analyzed based on the individual phylum of macroalgal species due to nonhomogeneity within group dispersion.

10.1128/mSphere.00665-21.3 FIG S3 Hierarchical clustering of macroalgal-associated microbiota communities using Euclidean distances. Download FIG S3, PDF file, 0.3 MB. Copyright © 2021 Kuba et al. 2021 Kuba et al. https://creativecommons.org/licenses/by/4.0/ This content is distributed under the terms of the Creative Commons Attribution 4.0 International license.

10.1128/mSphere.00665-21.10 TABLE S6 PERMANOVA results based on Bray-Curtis dissimilarities of amplicon sequence variant abundances for bacterial communities within the complete microbiota and macroalgal group-level microbial counterparts. An asterisk indicates a significant associated *P* value; this value indicates a difference between group dispersions in the betadisper test. Download Table S6, PDF file, 0.05 MB. Copyright © 2021 Kuba et al. 2021 Kuba et al. https://creativecommons.org/licenses/by/4.0/ This content is distributed under the terms of the Creative Commons Attribution 4.0 International license.

According to PERMANOVA, host phylum, species, and thallus complexity were significant factors in structuring the macroalga-associated microbiota (Table 3). No significant difference was identified between the four native and one invasive macroalgal species (*P* = 0.052) (Table 3). Calcification level was nonhomogeneous; therefore, analysis was performed on host phylum for the chlorophytes and ochrophytes. This analysis could not be conducted on the Rhodophyta, because this phylum was represented by one species. No significance was identified for these two phyla based on calcification level (Table S3).

## DISCUSSION

To better understand the microbial diversity of the entire alga under the same environmental constraints, macroalgae were collected at one time point and analyses were completed without separation of tissue specific regions. This showed distinct microbial communities based on host species and thallus characteristics. The microbial communities identified were typical of those associated with marine macroalgal species (Fig. 2) (56–58); however, they may vary based upon environmental conditions which were not measured in this study (54, 59–61). Abiotic stressors can impact the microbial community structure of multiple species within a single intertidal bench (62). These distinct relationships were driven by both phylogenetic and functional divisions of the macroalgae and also have been apparent in coral-associated microbial communities of the Great Barrier Reef and the Hawaiian Archipelago (63, 64).

The presence of *Verrucomicrobiota*, *Actinobacteriota*, *Bdellovibrionota*, *Bacteroidota*, and *Myxococcota* in association with abundant macroalgal species at ‘Ewa Beach suggests that these communities may be exposed to anthropogenically altered physicochemical water conditions (54, 55, 65–67). *Bdellovibrionota* has been associated with sewage and sewage-polluted waters, reproducing only in aerobic environments (68). Potential nitrogen-fixing bacteria present in association with macroalgal assemblages (i.e., *Verrucomicrobiota*, *Alphaproteobacteria*, and *Cyanobacteria*) increase in response to bioavailable nitrogen (69). The diversity in energy sources that these bacterial communities utilize may increase the strength of these macroalgal-microbial relationships, especially in response to certain environmental fluctuations (70, 71). While drainage pipes have been suggested as a source of nutrients in the ‘Ewa Beach area (38, 64), more recent studies found this effect to be limited to areas close to the pipes (38). The site selected in this study was not adjacent to the drainage pipes examined by Cox and Foster (38) and was similar in algal species composition to other intertidal environments in ‘Ewa (21). Additional spatial and temporal sampling is necessary to determine if these bacteria are present in association with macroalgae at other sites in the Hawaiian Archipelago with differing levels of natural (e.g., groundwater) and anthropogenic impacts.

Microbial communities can be distinct based on whether a species is native or invasive within a specific environment, further influencing the invasion capacity of the host (3, 45). The Executive Summary of the National Invasive Species Management Plan (NISMP) defines the term “invasive species” as “a species that is nonnative to the ecosystem under consideration and whose introduction causes or is likely to cause economic or environmental harm or harm to human health.” However, the microbial community of the invasive *A. lacerata* was not significantly different from that of *H. discoidea* (Chlorophyta) or *A. taxiformis* (Rhodophyta), showing that while host abundance may be influenced by anthropogenic activities, the microbial communities were influenced by the host phylum on this intertidal bench at the time of sampling. Interestingly, *A. taxiformis* is considered invasive in other ecosystems and possesses a high invasive risk in both tropical and temperate systems through range expansion (65, 66). The *A. taxiformis* samples used in this study may be invasive in origin (72), which may explain some of the overlap between the Rhodophyta and Chlorophyta communities. Finding no significant difference between microbial counterparts of invasive and native macroalgae supports a stronger influence of phylum on these microbial communities associated with this intertidal bench. However, *A. lacerata* may be more influenced by the microbial community on the native algae rather than the intrinsic host factors, although the influence of this association under different environmental variables, such as increased water temperatures and nutrient loading, should be examined. Future studies should also include a variety of other native and invasive species replicated spatially across multiple sites.

Both Ochrophyta species, *P. sanctae-crucis* and *D. sandvicensis*, had microbial community assemblages that were mostly distinct from those of the Rhodophyta and Chlorophyta. Ochrophyta are characterized by their own unique phytochemical profile, strongly attributed to high concentrations of phlorotannins and terpenes (73, 74). Phlorotannins play an integral role in ecosystem structure and function, specifically influencing microbial infection (73–75). The majority of identified natural products produced by Ochrophyta are associated with *Dictyota* spp. (76). It is therefore likely the production of secondary metabolites by the Ochrophyta that impacts the associated microbiota.

Thallus complexity of the host also influenced the associated microbial communities at ‘Ewa Beach. Thallus characteristics are key to the functional role for the macroalgae, and their development may also be directly impacted by associated microbes (12, 77). Bacterial communities can experience a functional shift through algal life history (78), specific host identity, evolutionary history, and morphological complexity (13); therefore, these differences between hosts were expected. At ‘Ewa Beach, the thallus complexity of the host strongly influenced the community composition of the associated microbiota.

These results provide insight into microbial separation based on macroalgal phylum, host species, and thallus complexity. By examining the entire host-associated microbiota from one location at a single time point, this study demonstrates the functional role of macroalgal hosts in influencing their associated microbiota and provides the first description of distinct bacterial communities associated with intertidal macroalgae at one site in ‘Ewa Beach in Hawai’i. Moreover, bacterial communities may influence macroalgal host phylogeny and thallus complexity. Future studies should focus on spatial and temporal comparisons of these macroalgal assemblages to identify the stability of the associated bacterial communities and the influence of anthropogenic nitrogen sources on these assemblages. Furthermore, to determine how microbes impact establishment of invasive algae, additional invasive species should be included in future studies. Collection and analysis of isotope data in future studies may elucidate the drivers of potential nitrogen-fixing bacteria associated with macroalgae. The identification of environmental drivers affecting these relationships, such as temperature and solar irradiance, and the connectivity between ecosystem types in the near future may also reveal connections related to macroalgal and ecosystem health, biodiversity, and overall community composition.

## MATERIALS AND METHODS

### Study site.

The study was conducted on an intertidal bench at ʻEwa Beach (21.3058 N, 158.0284 W) on the southwest shore of Oʻahu, HI (Fig. 1A). This intertidal zone is characterized by an intertidal bench that was described previously (21). The climate near this sample location is characterized by alternating wet and dry seasons with a fairly constant air temperature (21). The mean seasonal precipitation when these samples were collected ranged from 1.0 to 1.6 cm, and the mean air temperature ranges from ∼23.1 to 27.3°C (temperature and rainfall data retrieved from the nearest National Climatic Data Center, NOAA, at the ‘Ewa Kalaeloa Airport Station) (21).

### Sample collection.

Samples (3 each) of *Padina sanctae-crucis* Børgesen, *Dictyota sandvicensis* Sonder, *Halimeda discoidea* Decaisne, *Avrainvillea lacerata* J. Agardh, and *Asparagopsis taxiformis* (Delile) Trevisan (Fig. 1; Table 1) were collected on 19 May 2019 and identified visually using references 52, 53, and 79. Samples were collected at low tide (−0.27 ft) at 09:30 Hawaii-Aleutian Standard Time. Each sample was rinsed with 3.5% sterile artificial seawater to remove loosely attached epibionts and sand. Individual rinsed thalli (∼0.5 g) were then placed in RNAlater and stored overnight at 4°C before freezing at −80°C. Background seawater samples (*n* = 3) were collected by filtering 50 ml of seawater (80) through a sterile 0.2-μm filter and preserved in 5 ml of RNAlater. This volume of seawater has an associated average extraction efficiency of 92% for marine planktobacteria (80). Filters were stored at 4°C before freezing at −80°C. Voucher specimens were deposited at the Herbarium Pacificum, Bernice. P. Bishop Museum, Honolulu, HI (BISH) (Table S1).

10.1128/mSphere.00665-21.5 TABLE S1 Accession data for collected macroalgal specimens used in this study. Download Table S1, PDF file, 0.06 MB. Copyright © 2021 Kuba et al. 2021 Kuba et al. https://creativecommons.org/licenses/by/4.0/ This content is distributed under the terms of the Creative Commons Attribution 4.0 International license.

### DNA extraction.

All samples were thawed on ice and DNA was extracted from individual thalli using the FastDNA Spin kit (MP Biomedicals, Santa Ana, CA) following the manufacturer’s protocol with minor modifications. Approximately 0.5 g of each algal sample was weighed out into the lysis matrix E tube using ethanol flame-sterilized forceps. Thalli were split into two lysis tubes if an individual had a mass of >0.5 g. Each seawater control filter was cut and divided into lysis matrix E tubes to a final weight of 0.5 g. Extractions were completed on entire algal individuals to include both associated epibionts and endobionts. Lysis tubes were placed in a cold aluminum rack and homogenized at 3,800 rpm for 30 s (BioSpec BeadBeater). Bead beating was repeated twice with an incubation period of 30 s on ice between homogenizations. DNA was eluted twice with 50 μl 0.1 mM Tris (pH 8.0). DNA was quantified with a Qubit 3.0 fluorometer using the double-stranded-DNA (dsDNA) high-sensitivity kit (Thermo Fisher Scientific). The seawater controls were pooled prior to indexing and sequencing due to low DNA recovery.

### Amplification and sequencing.

PCR was performed using primers 340F (5′-CCTACGGGNGGCWGCAG-3′) and 784R (5′-GGACTACHVGGGTATCTAATCC-3′) (81) targeting the V3-V4 variable regions of the bacterial SSU rRNA gene that also possessed Illumina overhang sequences used for ligation of index sequences for all 15 macroalgal samples and the pooled seawater control. Cycling conditions were as follows: 95°C for 5 min, 25 cycles of 95°C for 45 s, 52°C for 45 s, and 72°C for 3 min, followed by a final extension at 72°C for 7 min. A post-PCR cleanup was completed using AMPure bead purification according to the manufacturer’s instructions (Beckman Coulter, Brea, CA). Cleaned amplicons were sent to the Medical University of South Carolina (Charleston, SC) for indexing and sequencing on an Illumina MiSeq per the manufacturer’s protocol, generating 2 × 300-bp paired-end reads.

### Sequence and statistical analysis.

Demultiplexed sequences with adapters removed were analyzed for quality using FastQC (82). Forward and reverse primers were trimmed from each sequence using CutAdapt v1.8 (83). Amplicon sequence variants were identified by the DADA2 (v1.18.0) package as implemented in R version 4.0.1 (R Development Core Team, 2010) as previously described (50, 84). Briefly, paired-end sequences were quality filtered to removed sequences with a quality score of <20. Sequences were dereplicated, and forward and reverse amplicon sequence variants (ASVs) were merged using a minimum overlap of 56 bp. Chimera identification and removal were performed using the “removeBimeraDenovo” command, and each phylum was assigned using the SILVA v138 reference database (85). The data were then transformed into a phyloseq (86) object for further analysis.

Statistical analyses were performed using R version 4.0.1 (R Development Core Team, 2010), and visualizations were generated using ggplot2 (87), phyloseq (86), and microbiome (88). Differential abundance analyses were utilized using the DESeq2 tool (89) with an alpha value of <0.01 to identify the ASVs contributing to the overall differences among samples. This tool accounts for low dispersion estimates and is consistent across studies with various replicates (89). Data were then normalized using a variance-stabilizing transformation (VST) to compare microbiota across samples. Samples were rarefied using the “rarecurve” command in the vegan R package (90) (Fig. S1). Visualizations of hierarchical clustering were performed with variance-stabilized Euclidean distances (Fig. S3). Alpha diversity was estimated using multiple indices (Table S2). The Simpson diversity index is used to compare the diversity of macroalga-associated microbiotas based on ASVs; values closer to 1 have lower microbial diversity. This diversity index was reported because of the greater weight it puts onto species evenness rather than richness, compared to the Shannon-Weaver index. Beta diversity was visualized using a nonmetric multidimensional scaling (NMDS) plot and the Bray-Curtis dissimilarity metric. Statistical significance to characterize the difference in microbial diversity across macroalgal group was determined using permutational multivariate analysis of variance (PERMANOVA) and the Bray-Curtis dissimilarity metric using the “adonis2” command in the vegan R package version 2.0-4 (90). Another PERMANOVA was used to determine statistical significance to characterize differences between macroalgal phylum and the background water control. Taxonomic distributions of macroalgal-associated microbiota were visualized using a bubble plot and a heat map through ggplot2 (87). The cutoffs for ASV abundance were set to greater than 0.5% in more than three samples.

### Data availability.

Sequence data are available through NCBI Sequence Read Archive (SRA) under study number SUB10020990 (BioProject number PRJNA748089).
