# Supplementary material for: Microbiota-Macroalgal Relationships at a Hawaiian Intertidal Bench Are Influenced by Macroalgal Phyla and Associated Thallus Complexity
Source: mSphere. 2021 Sep 22;6(5):e00665-21. doi: 10.1128/mSphere.00665-21 (PMC8550217; doi:10.1128/mSphere.00665-21)
Supplement: FIG S2 [file msphere.00665-21-sf002.pdf]

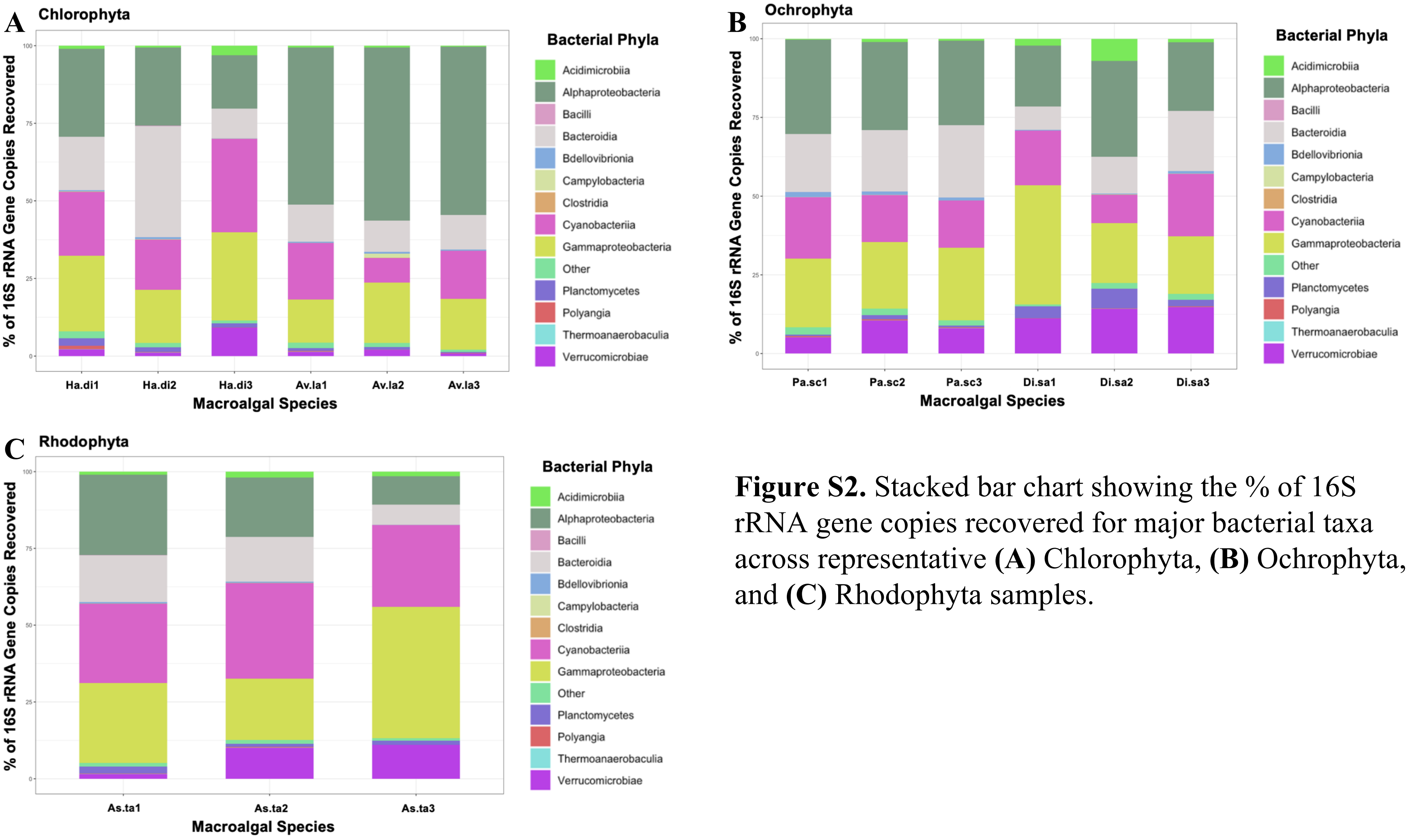

Figure S2. Stacked bar chart showing the % of 16S rRNA gene copies recovered for major bacterial taxa across representative **(A)** Chlorophyta, **(B)** Ochrophyta, and **(C)** Rhodophyta samples.
