# Supplementary material for: Microbiota-Macroalgal Relationships at a Hawaiian Intertidal Bench Are Influenced by Macroalgal Phyla and Associated Thallus Complexity
Source: mSphere. 2021 Sep 22;6(5):e00665-21. doi: 10.1128/mSphere.00665-21 (PMC8550217; doi:10.1128/mSphere.00665-21)

Figure S4. Heat map of the taxonomic distribution of macroalgal-associated bacteria genera of the top 3% in relative abundance**.** The relative abundance of bacteria phyla are provided for each sample. Each macroalgal phylum is considered (Ochrophyta, Chlorophyta, Rhodophyta), as well as the background seawater control. Each sample (n = 15) is shown except for seawater control samples (n = 3) which were pooled prior to indexing and sequencing. Sample identification corresponds to the given species: Pa.sa (*Padina sanctae-crusis*), Di.sa (*Dictyota sandvicensis*), Ha.di (*Halimeda discoidea*), Av.la (*Avrainvillea lacerata*), As.ta (*Asparagopsis taxiformis*), Wa (Seawater control).

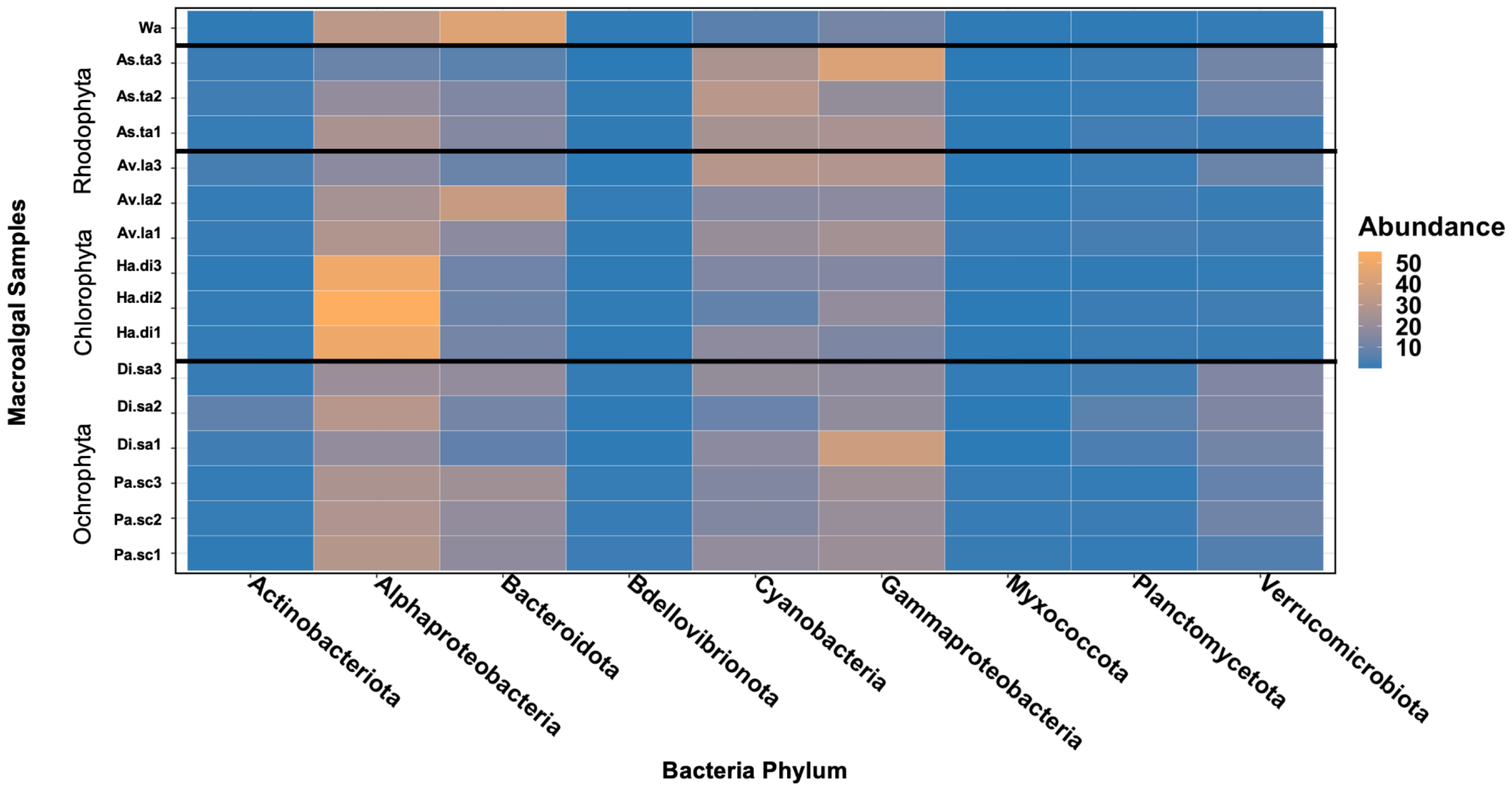

Supplement: FIG S4 [file msphere.00665-21-sf004.pdf]
