# Supplementary material for: Microbiota-Macroalgal Relationships at a Hawaiian Intertidal Bench Are Influenced by Macroalgal Phyla and Associated Thallus Complexity
Source: mSphere. 2021 Sep 22;6(5):e00665-21. doi: 10.1128/mSphere.00665-21 (PMC8550217; doi:10.1128/mSphere.00665-21)
Supplement: TABLE S3 [file msphere.00665-21-st003.pdf]

Table S3. Read counts throughout input, trimming, quality check, and chimera identification for each of the samples.

	DADA2_Input	Filtered Reads	DADA_Forward	DADA_Reverse	Merged Reads	No Chimera	Final % Reads Retained
**Pa.sc1**	525610	243683	236355	240636	219924	214464	40.8
**Pa.sc2**	586049	321271	313714	318451	296306	291021	49.7
**Pa.sc3**	527039	296407	292646	294811	282558	279616	53.1
**Di.sa1**	475816	253310	250817	252146	243124	236808	49.8
**Di.sa2**	571818	315994	311807	313498	299952	295935	51.8
**Di.sa3**	658678	371149	366072	368928	352900	346824	52.7
**Ha.di1**	378100	162205	156039	158738	144081	138653	36.7
**Ha.di2**	467101	230587	223194	227077	207361	187409	40.1
**Ha.di3**	743106	365461	352638	360556	318011	270189	36.4
**Av.la1**	1297700	665501	608833	645681	485489	451323	34.8
**Av.la2**	652174	344567	325845	336949	284840	258155	39.6
**Av.la3**	380693	192528	183554	189335	160019	139851	36.7
**As.ta1**	217148	104966	95946	101518	79225	75100	34.6
**As.ta2**	483233	246196	226981	239735	189290	169989	35.2
**As.ta3**	437519	212565	205867	209829	188724	165412	37.8
**Wa**	339494	120918	117915	119169	112985	112484	33.1
