# Supplementary material for: Microbiota-Macroalgal Relationships at a Hawaiian Intertidal Bench Are Influenced by Macroalgal Phyla and Associated Thallus Complexity
Source: mSphere. 2021 Sep 22;6(5):e00665-21. doi: 10.1128/mSphere.00665-21 (PMC8550217; doi:10.1128/mSphere.00665-21)
Supplement: FIG S1 [file msphere.00665-21-sf001.pdf]

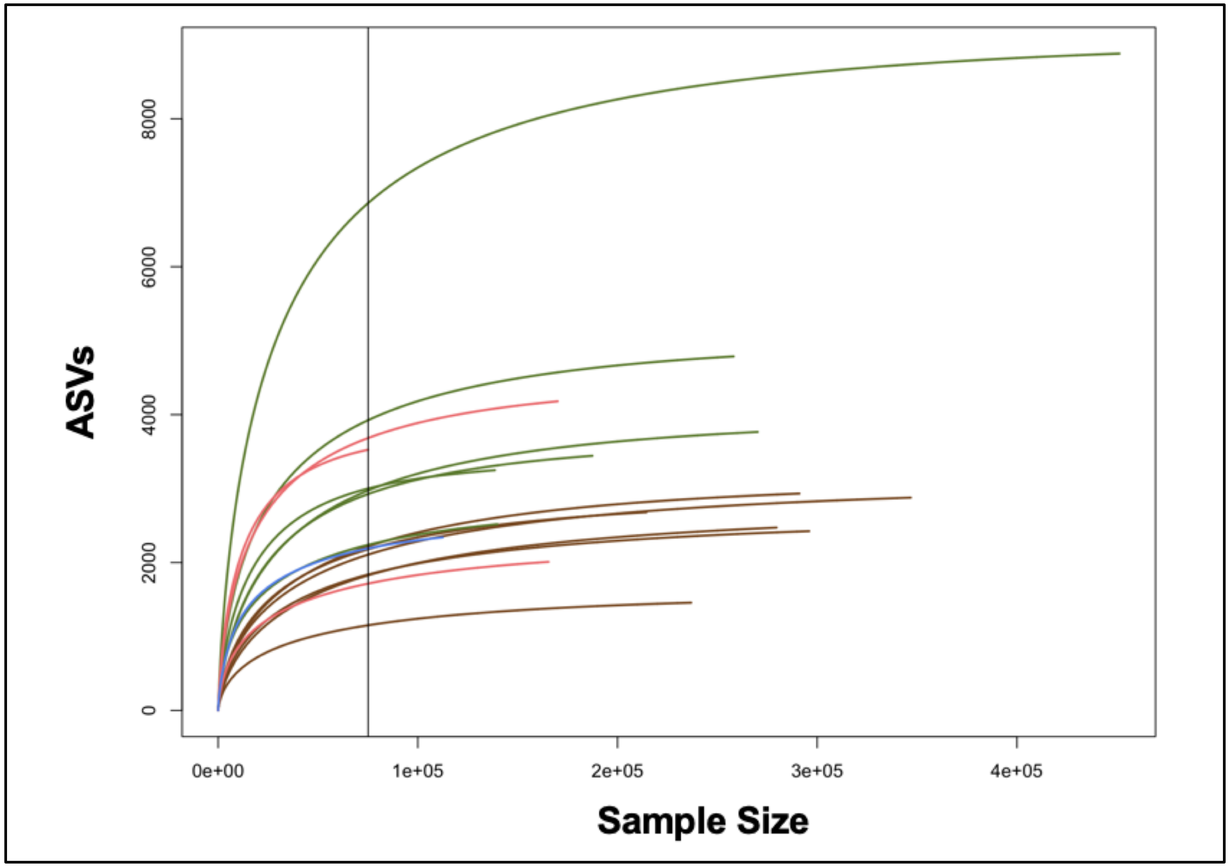

Figure S1. Rarefaction curves of bacterial partial small subunit (SSU) rRNA gene sequences for macroalgal samples and combined water control. Macroalgal phyla indicated by colors: Chlorophyta (green), Ochrophyta (brown), Rhodophyta (red). Water control (blue) is also included. Sampling depth associated with the fewest number of sequences indicated by the black line.
