# Supplementary material for: Microbiota-Macroalgal Relationships at a Hawaiian Intertidal Bench Are Influenced by Macroalgal Phyla and Associated Thallus Complexity
Source: mSphere. 2021 Sep 22;6(5):e00665-21. doi: 10.1128/mSphere.00665-21 (PMC8550217; doi:10.1128/mSphere.00665-21)
Supplement: TABLE S4 [file msphere.00665-21-st004.pdf]

Table S4. PERMANOVA (permutational analysis of variance) results based on Bray-Curtis dissimilarities of amplicon sequence variant abundances for bacterial communities within the complete macroalgal microbiota. Comparisons were made between microbial counterparts associated with specific macroalgal phyla. *significant associated p-value; this value indicates a difference in between group dispersion in the bestadisper test.

	**Sum of Squares**	**Mean Square**	**F-value**	**R^2**	**P-value**
**Chlorophyta: Rhodophyta**	104425	104425	1.1212	0.13806	0.292
**Rhodophyta: Ochrophyta**	159376	159376	2.553	0.26724	0.013*
**Ochrophyta: Chlorophyta**	228067	228067	2.853	0.22197	0.004*
**Ochrophyta: Water Control**	260762	260762	3.4264	0.40663	0.113
