# Supplementary material for: Microbiota-Macroalgal Relationships at a Hawaiian Intertidal Bench Are Influenced by Macroalgal Phyla and Associated Thallus Complexity
Source: mSphere. 2021 Sep 22;6(5):e00665-21. doi: 10.1128/mSphere.00665-21 (PMC8550217; doi:10.1128/mSphere.00665-21)
Supplement: TABLE S2 [file msphere.00665-21-st002.pdf]

Table S2. Observed amplicon sequence variants (ASVs) of bacterial taxa diversity indices of the microbial communities associated with macroalgal species. Diversity indices calculated include: Chao1, Abundance-based coverage estimator (ACE), Shannon-Weiner, Simpson, Inverse Simpson (InvSimpson), and Fisher.

	**Observed**	**Chao1**	**ACE**	**Shannon**	**Simpson**	**InvSimpson**	**Fisher**
**Pa.sc1**	2680	2879.59	2857.63	2.65	0.53	2.14	431.52
**Pa.sc2**	2932	3082.90	3078.75	2.64	0.56	2.27	453.46
**Pa.sc3**	2472	2679.23	2651.01	1.88	0.38	1.62	373.43
**Di.sa1**	1457	1556.75	1563.10	2.17	0.52	2.07	206.85
**Di.sa2**	2424	2567.05	2564.96	2.73	0.58	2.38	361.28
**Di.sa3**	2877	3055.73	3029.15	2.94	0.66	2.91	429.75
**Ha.di1**	3248	3380.53	3362.21	5.35	0.98	41.92	595.45
**Ha.di2**	3443	3615.68	3618.77	4.95	0.97	31.10	598.87
**Ha.di3**	3766	3936.45	3926.58	5.12	0.97	34.83	619.36
**Av.la1**	8882	9037.36	9043.71	7.18	0.99	138.89	1567.56
**Av.la2**	4787	4967.91	4966.00	5.73	0.97	36.69	834.26
**Av.la3**	2518	2730.20	2752.00	4.88	0.93	14.53	436.13
**As.ta1**	3525	3713.23	3734.88	6.33	0.99	87.12	767.34
**As.ta2**	4180	4387.51	4392.20	5.76	0.96	25.60	774.71
**As.ta3**	2009	2174.28	2200.09	3.69	0.83	5.77	321.73
**Wa**	2340	2494.55	2533.54	5.34	0.98	58.67	417.94
