# Supplementary material for: Microbiota-Macroalgal Relationships at a Hawaiian Intertidal Bench Are Influenced by Macroalgal Phyla and Associated Thallus Complexity
Source: mSphere. 2021 Sep 22;6(5):e00665-21. doi: 10.1128/mSphere.00665-21 (PMC8550217; doi:10.1128/mSphere.00665-21)
Supplement: TABLE S5 [file msphere.00665-21-st005.pdf]

Table S5. The top five most abundant bacterial genera associated with each macroalgal phylum (Ochrophyta, Rhodophyta, Chlorophyta) at ‘Ewa Beach, Hawai‘i, USA.

	**Macroalgal Phyla**		
**Top Bacterial Taxa**	**Ochrophyta**	**Rhodophyta**	**Chlorophyta**
**1**	Rivularia PCC-7116	Acrophormium PCC-7375	Rivularia PCC-7116
**2**	Hyphomonas	Hyphomonas	Hyphomonas
**3**	Litorimonas	Rivularia PCC-7116	Schizothrix LEGE 07164
**4**	Schizothrix 07164	Cognatishimia	Acrophormium PCC-7375
**5**	Acrophormium PCC-7375	Schizothrix LEGE 07164	Limibaculum
