# Supplementary material for: Microbiota-Macroalgal Relationships at a Hawaiian Intertidal Bench Are Influenced by Macroalgal Phyla and Associated Thallus Complexity
Source: mSphere. 2021 Sep 22;6(5):e00665-21. doi: 10.1128/mSphere.00665-21 (PMC8550217; doi:10.1128/mSphere.00665-21)
Supplement: FIG S3 [file msphere.00665-21-sf003.pdf]

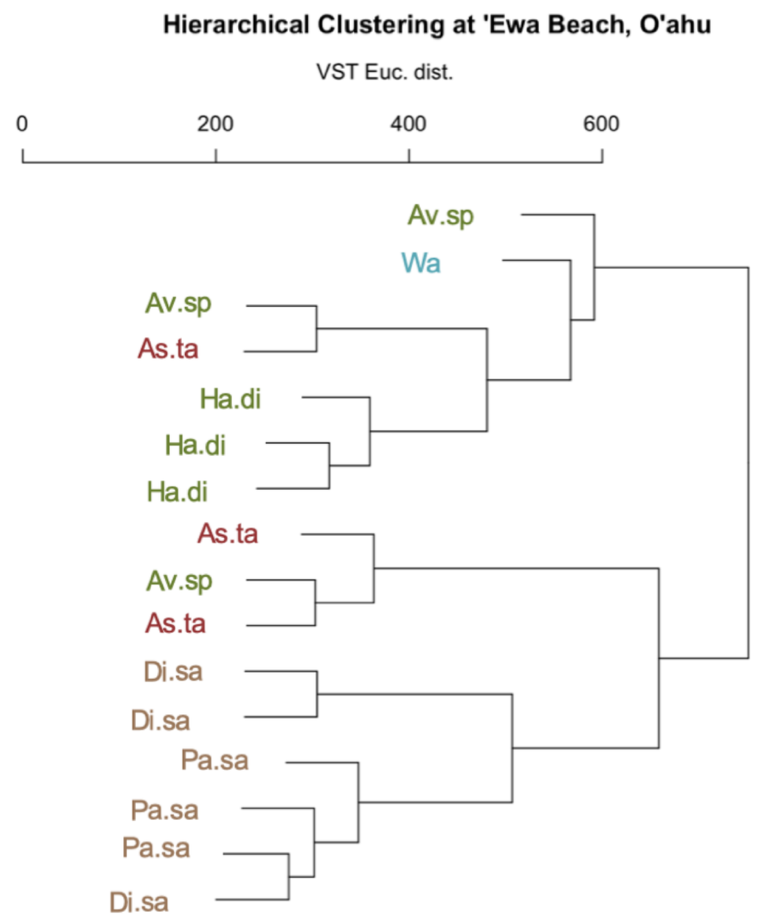

Figure S3. Hierarchical clustering of macroalgal-associated microbiota communities using Euclidean distances.
