# Supplementary material for: Microbiota-Macroalgal Relationships at a Hawaiian Intertidal Bench Are Influenced by Macroalgal Phyla and Associated Thallus Complexity
Source: mSphere. 2021 Sep 22;6(5):e00665-21. doi: 10.1128/mSphere.00665-21 (PMC8550217; doi:10.1128/mSphere.00665-21)
Supplement: TABLE S6 [file msphere.00665-21-st006.pdf]

Table S6: PERMANOVA (permutational analysis of variance) results based on Bray-Curtis dissimilarities of amplicon sequence variant abundances for bacterial communities within the complete microbiota and macroalgal group-level microbial counterparts. * significant associated p-value; this value indicates a difference in between group dispersion in the betadisper test.

**Factor**	**Sums of Squares**	**Mean Square**	**F-value**	**P-value**
Phylum	15153	7576.6	1.8934	0.1929
Host Species	26862	6715.4	2.2075	0.1413
Invasive/Native	1084.6	1084.6	0.4632	0.5081
Calcification Level	35103	17551.6	4.8193	0.02909*
Thallus Complexity	36973	12324.2	2.4583	0.1176
