# Supplementary material for: Microbiota-Macroalgal Relationships at a Hawaiian Intertidal Bench Are Influenced by Macroalgal Phyla and Associated Thallus Complexity
Source: mSphere. 2021 Sep 22;6(5):e00665-21. doi: 10.1128/mSphere.00665-21 (PMC8550217; doi:10.1128/mSphere.00665-21)
Supplement: TABLE S1 [file msphere.00665-21-st001.pdf]

Table S1. Accession data for collected macroalgal specimens used in this study.

Species	Voucher Number	Bishop Accession Number
Avrainvillea lacerata	GMK59	783320
Asparagopsis taxiformis	GMK57	783319
Dictyota sandvicensis	GMK58	783318
Halimeda discoidea	GMK53	783316
Padina sanctae-crucis	GMK56	83315
